# Multidisciplinary management of Grade III circumferential mixed hemorrhoids in a patient with Marfan syndrome receiving long-term anticoagulation: a case report

**DOI:** 10.3389/fsurg.2026.1846705

**Published:** 2026-06-15

**Authors:** Baopei Wei, Xianzhe Wu, Yirong Zhang

**Affiliations:** 1 Department of Anorectal Surgery, Shenzhen Luohu People’s Hospital, Shenzhen, Guangdong, China; 2 Department of Pharmacy, Shenzhen Kangning Hospital, Shenzhen, Guangdong, China

**Keywords:** case report, enteral nutrition, hemorrhoids, long-term anticoagulation, Marfan syndrome, multidisciplinary team, perioperative management

## Abstract

Management of advanced hemorrhoidal disease in patients with Marfan syndrome (MFS) who require lifelong anticoagulation is clinically complex because perioperative care must balance prosthetic valve thrombosis against postoperative anorectal bleeding. We report the case of a 33-year-old male with MFS, status post-aortic dissection repair (Sun's procedure and Bentall operation) on continuous warfarin therapy, who presented with persistent Grade III circumferential mixed hemorrhoids that had not responded adequately to prior conservative outpatient management. Physical examination confirmed characteristic MFS musculoskeletal signs and severe hemorrhoidal prolapse. A structured multidisciplinary team (MDT) pathway involving colorectal surgery, cardiovascular medicine, vascular surgery, anesthesia, pharmacy, and nutrition was used to individualize perioperative management. After cardiology consultation, and based on the patient's high thromboembolic risk, preoperative warfarin interruption was shortened to 3 days with enoxaparin bridging, and the INR decreased to 1.01 on the day before surgery. Rubber band ligation was avoided because of concern for delayed bleeding after sloughing in an anticoagulated patient. Because the disease involved circumferential mixed hemorrhoids with a prominent external component and prolapse, repeated sclerotherapy alone was considered unlikely to provide adequate definitive control. The patient therefore underwent external dissection and internal ligation combined with liquid polidocanol injection sclerotherapy, with minimal intraoperative blood loss. Postoperatively, a 25-day inpatient observation period enabled close INR titration during warfarin reinitiation, controlled bowel management, staged low-residue enteral nutrition, avoidance of rectal suppositories, and direct monitoring for delayed bleeding. Following minor self-limiting hematochezia, the patient was discharged with an INR of 1.8 as a pragmatic compromise between bleeding and thrombotic risks. At 41 months of follow-up, the wounds remained well healed, with no recurrent prolapse or severe hemorrhagic complications. This case suggests that surgical intervention for advanced hemorrhoids in anticoagulated MFS patients may be feasible when embedded within a structured, individualized multidisciplinary pathway. The anticoagulation, nutritional, and inpatient-monitoring strategies described here should be interpreted as patient-specific measures requiring further validation.

## 1 Introduction

Marfan syndrome (MFS) is an autosomal dominant connective tissue disorder characterized by cardiovascular, skeletal, and ocular manifestations (1). Patients with MFS may require complex cardiovascular surgery, including aortic dissection repair and composite valve-graft procedures, followed by lifelong anticoagulation when a mechanical prosthesis is implanted. Although hemorrhoidal disease is common (2), the management of Grade III circumferential mixed hemorrhoids in a patient requiring continuous anticoagulation remains clinically challenging. In this setting, definitive anorectal treatment must be weighed against the competing risks of prosthetic valve thrombosis and postoperative hemorrhage. This report describes the successful surgical management of such a case, with emphasis on structured multidisciplinary planning, individualized perioperative anticoagulation, procedure selection, bowel management, and long-term follow-up.

## 2 Case description

### 2.1 Patient information

A 33-year-old male with a confirmed diagnosis of MFS, status post-aortic dissection repair (Sun's procedure and Bentall operation) on continuous warfarin therapy, presented to our department. His main concern was persistent prolapse and discomfort related to Grade III circumferential mixed hemorrhoids. Before referral, the patient had attempted conservative outpatient measures, including dietary and bowel-habit adjustment and topical anti-hemorrhoidal therapy, but the prolapse persisted and continued to affect daily activities. No previous procedural hemorrhoid treatment, such as rubber band ligation, sclerotherapy, or hemorrhoidectomy, was documented before admission. Family history revealed that the patient's father had died from aortic dissection associated with MFS, supporting an autosomal dominant inheritance pattern (3). The patient expressed anxiety regarding both thrombotic risk from his cardiovascular history and bleeding risk during anorectal surgery, which was addressed during perioperative counseling.

### 2.2 Clinical findings

**Figure 1:**
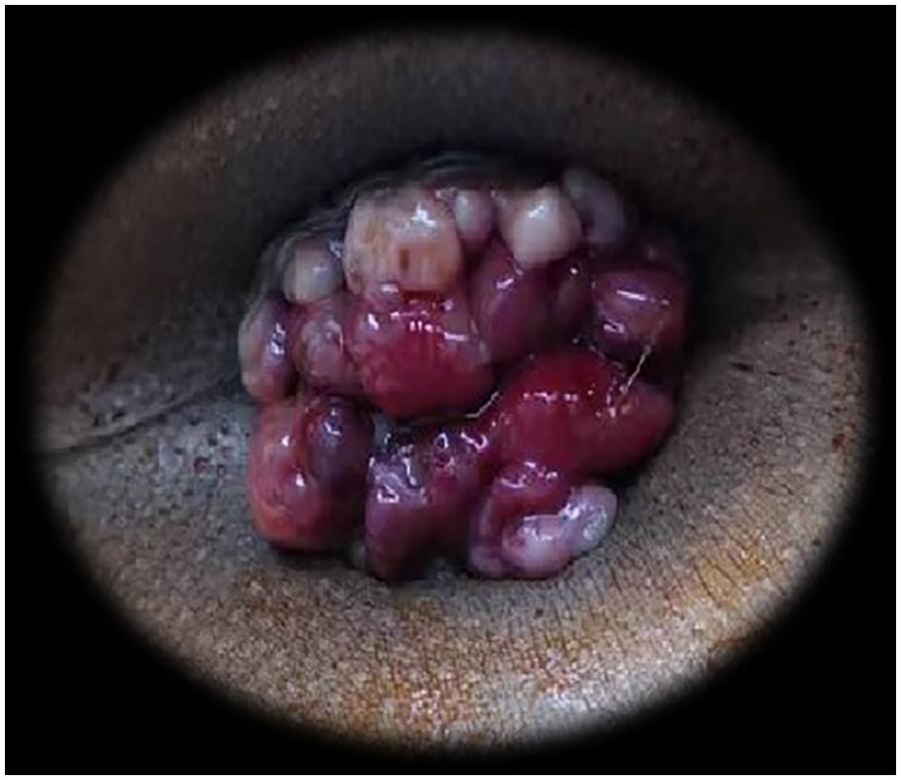
Preoperative external anal examination showing severe prolapse of grade III circumferential mixed hemorrhoids beyond the anal verge, readily visible without the aid of an anoscope.

Physical examination demonstrated characteristic musculoskeletal manifestations of MFS, including a positive thumb (Steinberg) sign and a positive wrist (Walker-Murdoch) sign. External anal examination showed severe prolapse of Grade III circumferential mixed hemorrhoids beyond the anal verge, which was readily visible without anoscopy (Figure 1). No other concomitant anorectal lesions were identified.

### 2.3 Timeline

The patient was admitted 3 days before surgery. Warfarin was discontinued on admission and bridging anticoagulation was initiated after cardiovascular assessment. Surgical intervention was performed on day 0 (October 20, 2022). Oral warfarin was resumed on postoperative day 2. A staged low-residue liquid diet was maintained from postoperative days 1 to 14, followed by gradual transition to a semi-liquid diet on postoperative day 15. After a 25-day inpatient observation period focused on INR titration, bowel management, wound monitoring, and dietary supervision, the patient was discharged. Long-term outpatient follow-up was subsequently maintained. The clinical timeline is summarized in Table 1.

**Table 1:** CARE-compliant clinical timeline.

Date/period	Episode of care	Key details and outcome
Before admission	Relevant background	Confirmed MFS; prior aortic dissection repair with Sun's procedure and Bentall operation; continuous warfarin therapy; family history of MFS-associated aortic dissection; persistent Grade III circumferential mixed hemorrhoidal prolapse despite prior conservative outpatient measures.
3 days before surgery	Admission and anticoagulation planning	Warfarin discontinued; enoxaparin bridging initiated after cardiovascular assessment.
October 19, 2022	Preoperative INR assessment	INR decreased to 1.01 on the day before surgery.
October 20, 2022	Surgical intervention	External hemorrhoidectomy, internal hemorrhoid ligation, and liquid polidocanol injection sclerotherapy; RBL avoided; operative time 35 min; estimated blood loss 5 mL.
October 21, 2022	Early postoperative monitoring	INR was 0.92 on postoperative day 1.
Postoperative day 2	Warfarin reinitiation	Oral warfarin resumed with continued bridging according to inpatient monitoring.
Postoperative days 1–4	Nutritional and bowel management	Commercial enteral nutrition powder used as the main nutritional source to reduce fecal bulk and wound friction.
Postoperative days 5–14	Nutritional progression	Whey protein powder added according to tolerance; high-residue foods still avoided.
Postoperative day 15	Diet transition	Gradual transition to semi-liquid foods after absence of active wound bleeding, acceptable stool consistency, and stable bowel frequency.
Postoperative day 25	Discharge	Minor self-limiting hematochezia had resolved; discharged with INR 1.8 as a pragmatic compromise between bleeding and thrombotic risks.
January 13, 2023	Short-term follow-up	Well-healed anal wounds; no complications; INR 1.91.
December 19, 2025	Long-term follow-up at 38 months	One minor self-limiting hematochezia episode; follow-up photograph showed a well-healed site, mild venous congestion, no active bleeding or recurrent prolapse.
April 2, 2026	Most recent follow-up at 41 months	Patient remained asymptomatic and highly satisfied with the surgical outcome.

### 2.4 Diagnostic assessment

Given the patient's history of aortic dissection repair, mechanical valve replacement, and mandatory long-term warfarin therapy, a structured multidisciplinary team (MDT) pathway was established. In this report, MDT management refers to a pre-agreed interdisciplinary decision-making process rather than informal sequential consultation. The team included specialists from colorectal surgery, cardiovascular medicine, vascular surgery, anesthesia, pharmacy, and nutrition (4). Its functions included thrombotic and bleeding risk assessment, perioperative anticoagulation planning, selection of a bleeding-conscious anorectal procedure, nutritional and bowel-management planning, local wound-care precautions, serial INR review, and discharge-threshold determination. The central clinical dilemma was how to obtain durable hemorrhoidal control while minimizing both prosthetic valve thrombosis and postoperative surgical-site bleeding.

### 2.5 Therapeutic intervention

Preoperative Bridging: Upon admission, warfarin was discontinued and bridging therapy was initiated with subcutaneous enoxaparin sodium (Clexane, 4,000 IU according to local protocol). Most perioperative vitamin K antagonist (VKA) protocols use approximately 5 days of preoperative interruption to allow INR normalization; however, contemporary guidance emphasizes individualized assessment of thrombotic and bleeding risks, and recommendations regarding bridging in mechanical-valve patients are not entirely uniform across major guidelines (5, 6). The 2022 CHEST guideline suggests against routine heparin bridging in many patients with mechanical heart valves who require VKA interruption, whereas the 2020 ACC/AHA valvular heart disease guideline supports bridging in selected mechanical-valve patients with higher thromboembolic risk features (5, 6). In the present case, after formal cardiology consultation, the team chose a shortened 3-day warfarin interruption to reduce the duration of subtherapeutic anticoagulation while still permitting adequate perioperative hemostasis. This strategy was based on the patient's perceived high thrombotic risk, the availability of close inpatient monitoring, and the observed INR response. The INR decreased to 1.01 on the day before surgery (October 19) and was 0.92 on postoperative day 1 (October 21). Therefore, this approach should be interpreted as an individualized decision rather than a general recommendation for patients receiving warfarin.

Surgical Procedure: Under general anesthesia, the patient underwent definitive anorectal surgery. Rubber band ligation (RBL) was avoided because delayed secondary bleeding may occur when the ligated tissue sloughs, typically 7 to 14 days after the procedure, and this concern is particularly relevant in anticoagulated patients (7). Repeated sclerotherapy alone was also not selected as the initial definitive strategy. Although sclerotherapy can be useful for internal hemorrhoidal bleeding, this patient had Grade III circumferential mixed hemorrhoids with a prominent external component and persistent prolapse. Therefore, repeated sclerotherapy alone was considered unlikely to correct the external component or provide durable control of prolapse, and multiple sessions might have exposed this anticoagulated high-risk patient to repeated procedural bleeding risks. The MDT therefore selected a combined approach consisting of external hemorrhoidectomy, internal hemorrhoid ligation, and liquid polidocanol injection sclerotherapy. This was not polidocanol foam sclerotherapy. The combined strategy allowed direct hemostasis of the external components while using polidocanol injection to treat internal components without relying on delayed band sloughing. The operative time was 35 min, and estimated blood loss was 5 mL.

Postoperative Management: Bridging therapy with enoxaparin was reinitiated, and oral warfarin was resumed on postoperative day 2. To reduce fecal bulk and mechanical friction against the fresh anorectal wounds, the MDT implemented a staged low-residue enteral nutrition strategy. Because exact daily caloric intake, protein intake, and fiber quantity were not prospectively recorded, the nutritional regimen is reported by stage and clinical intent rather than as a formal quantitative prescription. During postoperative days 1–5, commercial enteral nutrition powder (Ensure) was used as the main nutritional source. During postoperative days 6–14, whey protein powder was added according to tolerance, while high-residue foods were still avoided. From postoperative day 15, the patient was gradually transitioned to semi-liquid foods such as congee and soft noodles after confirming the absence of active wound bleeding, acceptable stool consistency, and stable bowel frequency. Hemodynamic stability was supported with continuous daily oral metoprolol (Betaloc), and oral Aescin extract was administered to alleviate perianal edema. For local wound management, Titanoreine (compound titanium dioxide) cream was applied topically. Rectal suppositories were avoided to minimize mechanical irritation or iatrogenic trauma to the incompletely healed wounds.

### 2.6 Follow-up and outcomes

The prolonged postoperative hospitalization was primarily intended to support close INR titration during warfarin reinitiation, controlled bowel management, dietary supervision, and direct observation for delayed anorectal bleeding. Following enoxaparin bridging and resumption of warfarin, serial INR measurements showed a progressive rise. On postoperative day 25, the patient was discharged with an INR of 1.8 (Table 1), after a minor, self-limiting episode of hematochezia had resolved. This discharge INR represented a pragmatic compromise between the competing risks of recurrent wound bleeding and prosthetic valve thrombosis.

Short-term outpatient follow-up at approximately 3 months (January 13, 2023) showed well-healed anal wounds, no complications, and a stable near-therapeutic INR of 1.91.

**Figure 2:**
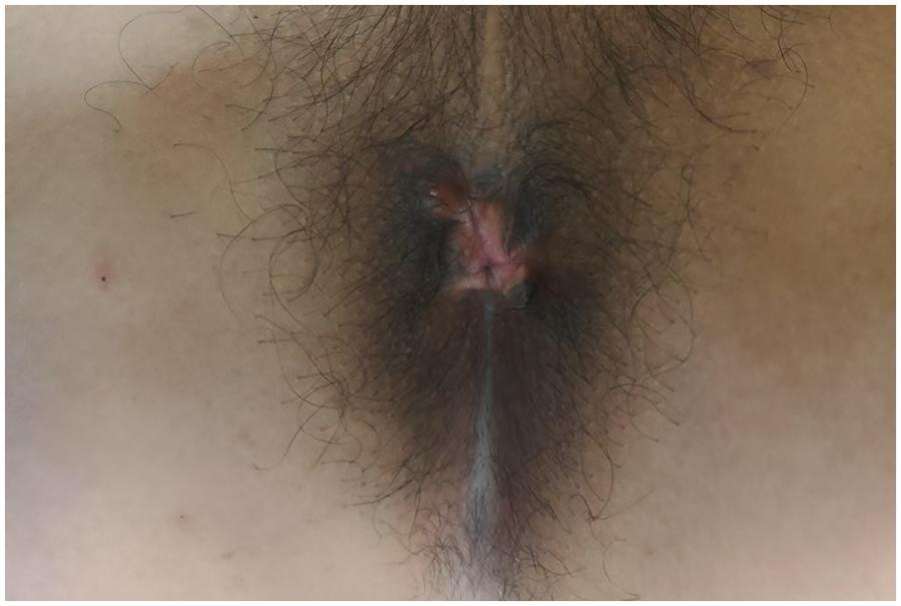
Long-term follow-up photograph at 38 months postoperatively (December 19, 2025). The perianal region shows a well-healed surgical site with normal anatomical contours. Mild hemorrhoidal venous congestion is visible, consistent with the patient's long-term anticoagulation status, but there is no evidence of recurrent hemorrhoidal prolapse or active mucosal hemorrhage.

The patient was followed longitudinally until April 2, 2026 (41 months postoperatively). During this extended period, anorectal function remained stable. On December 19, 2025 (38 months postoperatively), the patient reported a single episode of minor, self-limiting hematochezia. A follow-up photograph (Figure 2) showed a well-healed surgical site with mild hemorrhoidal venous congestion, but no active mucosal bleeding or recurrent prolapse. At the most recent follow-up on April 2, 2026, the patient remained asymptomatic and was highly satisfied with the surgical outcome.

## 3 Discussion

The management of Grade III mixed hemorrhoids in patients with MFS receiving long-term anticoagulation requires individualized balancing of thromboembolic and bleeding risks. The present case illustrates how a structured MDT pathway can integrate cardiovascular risk assessment, surgical modification, anticoagulation monitoring, bowel management, and wound-care precautions. The shortened 3-day warfarin interruption was deliberately individualized and should not be interpreted as evidence that a 3-day interruption is superior to the commonly used 5-day approach. Rather, it demonstrates that, in carefully selected patients, deviation from routine timing may be considered only after specialist cardiovascular consultation, serial INR monitoring, and explicit assessment of competing risks.

Procedure selection was central to risk reduction. RBL is widely used for hemorrhoidal disease, but delayed bleeding after tissue sloughing is a recognized concern, especially in patients receiving antithrombotic therapy (7). Sclerotherapy alone was not selected as a staged first-line procedural approach because the disease was not limited to internal hemorrhoidal bleeding. The patient had circumferential mixed hemorrhoids with an external component and clinically significant prolapse; therefore, repeated sclerotherapy alone was considered unlikely to provide sufficient anatomical correction or durable symptom control. In addition, repeated procedural sessions could have created multiple periods of bleeding risk while anticoagulation was being managed. Polidocanol foam injection sclerotherapy has been reported as a treatment option for mixed hemorrhoid bleeding in patients receiving antithrombotic therapy (8); however, the present case used liquid polidocanol injection rather than foam. Direct dissection-ligation combined with liquid polidocanol injection sclerotherapy provided an alternative strategy that allowed immediate hemostatic control of the external components while treating internal components without relying on delayed band necrosis. Avoidance of rectal suppositories was another practical wound-care modification, as mechanical friction from suppository insertion could theoretically disturb early wound healing in an anticoagulated patient.

Postoperative bowel management was a key element of the strategy. The staged low-residue enteral nutrition plan was intended to reduce stool volume and local trauma during the highest-risk period for wound bleeding. However, because exact caloric intake, protein intake, and fiber quantity were not prospectively documented, the nutritional intervention should be viewed as a clinically guided staged dietary approach rather than a reproducible quantitative nutrition protocol. Future reports or prospective studies should record daily intake, protein supplementation, stool frequency, stool form, and the criteria used for fiber reintroduction.

The 25-day hospitalization requires specific interpretation. It was not presented as a routine requirement for all similar patients. In this case, inpatient observation allowed daily or frequent INR assessment during warfarin reinitiation, direct monitoring for delayed bleeding, and strict bowel and dietary supervision while the anorectal wounds were vulnerable. In healthcare systems where prolonged admission is not feasible, an equivalent pathway may require scheduled outpatient INR testing, rapid access to colorectal review, explicit dietary instructions, and clear return precautions.

Strengths and limitations of this case: A major strength is the extended 41-month follow-up, which supports the durability of the surgical outcome in this patient despite lifelong anticoagulation. The minor self-limiting bleeding episode observed 38 months postoperatively required no dose adjustment or secondary intervention, and follow-up examination showed no recurrent prolapse. The principal limitation is the single-case design. The results cannot be generalized directly to all patients with MFS, mechanical valves, or long-term anticoagulation. In particular, the abbreviated warfarin interruption, the use of enoxaparin bridging, the combined dissection-ligation plus liquid polidocanol injection sclerotherapy technique, the staged low-residue nutritional pathway, and prolonged inpatient monitoring require prospective or multicenter validation before broader adoption.

## 4 Conclusion

This case suggests that surgical treatment of advanced hemorrhoidal disease may be feasible in selected anticoagulated patients with MFS when management is embedded within a structured multidisciplinary pathway. Individualized anticoagulation planning, avoidance of procedures associated with delayed bleeding, direct surgical hemostasis, careful wound care, staged bowel and dietary management, and long-term follow-up were central to the favorable outcome. Because this is a single case, the strategy should be considered hypothesis-generating and should not replace guideline-based individualized perioperative assessment.

## 5 Patient perspective

The patient expressed high satisfaction with the multidisciplinary care, noting that the tailored surgical approach and strict postoperative guidance alleviated his anxiety regarding both his cardiovascular condition and surgical recovery.

## Data Availability

The original contributions presented in the study are included in the article/supplementary material, further inquiries can be directed to the corresponding author.
